# Long-term effects of Garcinia *cambogia/*Glucomannan on weight loss in people with obesity, PLIN4, FTO and Trp64Arg polymorphisms

**DOI:** 10.1186/s12906-018-2099-7

**Published:** 2018-01-24

**Authors:** Andrea Maia-Landim, Juan M. Ramírez, Carolina Lancho, María S. Poblador, José L. Lancho

**Affiliations:** 1 Department of Morphological Sciences, School of Medicine, University of Córdoba, Avenida de Menéndez Pidal s/n, 14071 Córdoba, Spain; 2 Centro de parálisis cerebral Aspace, camino de Illarra s/n CP, 20018 Donostia/San Sebastián, Guipúzcoa Spain

**Keywords:** Overweight, Obesity, Polymorphisms

## Abstract

**Background:**

Overweight and obesity are considered major health problems that contribute to increase mortality and quality of life. Both conditions have a high prevalence across the world reaching epidemic numbers. Our aim was to evaluate the effects of the administration of Garcinia *cambogia* (GC) and Glucomannan (GNN) on long-term weight loss in people with overweight or obesity.

**Methods:**

Prospective, not-randomized controlled intervention trial was conducted. We treated 214 subjects with overweight or obesity with GC and GNN (500 mg twice a day, each) for 6 months evaluating weight, fat mass, visceral fat, basal metabolic rate, and lipid and glucose blood profiles comparing them with basal values. Some patients were carriers of polymorphisms PLIN4 -11482G > A-, fat mass and obesity-associated (FTO) -rs9939609 A/T- and β-adrenergic receptor 3 (ADRB3) -Trp64Arg**.**

**Results:**

Treatment produced weight loss, reducing fat mass, visceral fat, lipid and blood glucose profiles while increasing basal metabolic rate. Results were independent of sex, age or suffering from hypertension, diabetes mellitus type 2 or dyslipidemia and were attenuated in carriers of *PLIN4, FTO, Trp64Arg* polymorphisms.

**Conclusions:**

Administration of GC and GNN reduce weight and improve lipid and glucose blood profiles in people with overweight or obesity, although the presence of polymorphisms *PLIN4*, *FTO* and *ADRB3* might hinder in some degree these effects. ISRCTN78807585, 19 September 2017, retrospective study.

**Electronic supplementary material:**

The online version of this article (10.1186/s12906-018-2099-7) contains supplementary material, which is available to authorized users.

## Background

Obesity may be defined as abnormal or excessive fat accumulation that impairs health [1]. This illness is a global pandemic with a high prevalence in western societies that is also fast spreading across developed countries [1]. In 2008 was estimated that about 1.46 billion people suffer from overweight in the world and of them 504 million were people with obesity [2]. Obesity and its comorbidities represent a social-economic burden that reduce life expectancy and it has been associated with suffering from osteoarthritis, fatty liver, cardiovascular disease, digestive problems, diabetes mellitus type 2 (DM2) and even different kinds of cancer [2–4]. In childhood, in which overweight and obesity numbers are dramatically increasing, other pathologies such us orthopedic complications, breathing problems and psychological disorders have also been reported [5]. For practical reasons, overweight is defined as body mass index (BMI, weight in kg divided by height squared m^2^, also kwon as Quetelet’s index) between 25 and 30 and obesity when BMI > 30 [6].

The causes of obesity are several, although and increase in calories ingest together with sedentary way of life are intrinsically associated to its development being both of them preventable [7]. Other causes also linked to obesity are socioeconomic factors, birth weight, pollution, stress and microbial infections [8, 9]. Genetic background also plays a key role in obesity development [10]. Studies carried out in twins reported that obesity might be heritable component between 40 and 75% [11] and multiple genetics syndromes has been related to obesity [12]. Thus, some polymorphism present in genes such as Fat mass and obesity-associated (*FTO*), Perilipin (PLIN) and β-adrenergic receptor 3 (ADRB3) have been studied associated with obesity development. *FTO* is located in humans in chromosome 16 and codifies the enzyme 2-oxoglutarate nucleic acid dependent demethylase that is expressed in the hypothalamus and regulates food intake [13]. Variations of *FTO* gene has been associated to an increase in food intake [14]. In particular, polymorphism rs9939609 A/T has been association to regulation of satiety feeling [15]. On the other hand, it has been reported that *PLIN* gene (located in chromosome 15) and with several described polymorphism such as *PLIN1* 6209 T → C, *PLIN4* 11482G → A and *PLIN6* 14995A → T have been associated with obesity development or protection [16, 17]. Perilipin 1, is a protein present in adipocytes that play an important role in fat accumulation and mobilization, covering the lipids droplets protecting them from lipase enzymatic action [18]. In addition, *ADRB3* polymorphism Trp64Arg has been strongly associated to obesity and DM2 [19]. Activation of this receptor plays a role in lipolysis and thermogenesis regulation [15].

Different strategies have been proposed to deal with obesity epidemic. Caloric restriction combined with exercise are the most used one against this disease and in cases of extreme obesity, surgical intervention is recommended [20]. Other strategies include the use of herbal medicinal products from Garcinia *camboia* (GC) and Glucomannan that have reported to be positive in relation to control and loss weight [21–25]. GC is a fruit that naturally grows in South Asia and Indian forest with a high content of hidroxicitric acid (AHC). AHC inhibits lipogenesis impairing hydrocarbon conversion in lipids. AHC produces the inhibition of ATP-citrate liase, an enzyme that is required for the first step in lipogenesis process. AHC action also increments glycogen hepatic deposit, decrease appetite and reduces weight gain [26]. On the other hand, GNN is a fiber composed of β 1,4-linked D mannose and D-glucose monomers extracted from a tuber called *Amorphophallus konjac* [27]. In spite of GNN mechanism of action has not yet been fully understood, some authors have suggested, that given that GNN is able to absorb 50 times its weight in water volume, this fiber would fill up the stomach resulting in a delayed gastric emptying causing a satiety feeling and reducing the appetite [28].

In this work, we aimed to assess whether a controlled diet supplemented with GC and GNN was able to reduce weight after 3 and 6 moths in people with overweight or obesity. We also wanted to evaluate if this reduction was modified by the presence in the patients of different genetic polymorphism related to FTO (rs9939609 A/T), PLIN4 (11482G > A) and ADRB3 Trp64Arg.

## Methods

### Patients

This study was designed as a prospective, not-randomized controlled intervention trial to test differences in matched pairs. A Transparent Reporting of Evaluations with Nonrandomized Designs (TRENDS) checklist of the study and a flowchart diagram regarding subjects enrollment, follow-up, allocation and interventions have been included as supplementary material (Additional file 1: Table S1 and Additional file 2: Fig. S1). Patients from both sex and different ages were recruited from february 2015 to march 2017 (*n* = 214). All of them were above 18 years old and have an IMC > 25. We included subjects suffering dyslipidemias, hypertension, DM2 or their combinations. These patients were under treatment for hypertension, DM2 or dyslipidemias for a range of 2 months to 4 years before study onset. Exclusion criteria included: pregnancy or lactation, gastroplasty or gastrointestinal weight-reducing surgery, stopped smoking during the past 6 months, kidney disease, history of recurrent kidney stones, liver dysfunction, untreated high blood pressure, history or symptoms of gallstones, cancer, history of endocrine disorders (particularly hypothyroidism), history of bulimia and/or laxative abuse, mental disorders with impaired independence, history of alcohol or other drug abuse. Patient’s main characteristics are summarized in Table 1. All of them were evaluated at 3 and 6 months from study onset in Scientifics Aesthetics Clinics of the body from Córdoba, Sevilla and Huelva (Spain) or from Hospital e Maternidade São Francisco de Assis, Crato, (Brasil). All of them complete, previously to study onset, a form to evaluate their medical history.

**Table 1:** Subjects description by sex, age, BMI and polymorphism

Characteristic		Number	%
Sex	Male	122	57
	Female	99	43
BMI	25–30	125	58.41
	30–40	83	38.78
	> 40	6	2.80
Polymorphism	No “Normal”	138	64.48
	PLIN4 (11482G > A)	25	11.68
	FTO (rs9939609 A/T)	26	12.14
	ADRB3 (Trp64Arg)	25	11.68
Comorbidities	DM2	16	7.47
	H	42	19.62
	D	46	21.49
	H + D	52	24.29
	DM2 + H + D	58	27.10

DM2: Diabetes mellitus type 2, H: Hypertension arterial, D: dyslipidemias

### Pharmacological treatment

Patients were advised to have a balanced diet (Mediterranean diet), regular meals and intake of plenty of water. Standardized extracts of GC (52.4% HCA) and *A. konjac* (94.9%, Glucomannan) were administered separately in capsules of 500 mg each of them. We treated the patients with GC (500 mg), twice a day, half an hour before lunch and dinner and GNN (500 mg), twice a day, half an hour before lunch and dinner. It was recommended to patients to practice physical exercise, avoid smoking, and control alcohol intake.

### Anthropometric measurements

Anthropometric measurements and body composition: Body mass was measured on a digital balance (HD-305 TanitaTM) to the nearest 0.1 kg, and the height was measured with a Seca Bodymeter 206 to the nearest 0.1 cm. These data were used to calculate body mass index (BMI kg/m^2^).

Bioelectrical impedance was performed with a BioScan Spectrum operating at 50 KHz, measuring fat mass. Briefly, percentage fat mass (%FM) and fat free mass (FFM) were measured in 12 h fasted subjects on a restricted physical-activity schedule. FFM was assessed using the equation [29]: FFM (kg) =0.340 (h^2^/R) + 0.1534 (h) + 0.273 (BM) - 0.127 (age) + 4.56 (sex) - 12.44 where: h is the height (cm), R is the resistance (ohm) and female =0, male =1.

Basal Metabolic rate (BMR) was assessed by indirect calorimetry, using a TEEM 100® (INBRASPORT) calorimeter: 12 h fasted subjects on a restricted exercise schedule lay on their backs in a silent room at a mean temperature of 24 ± 1 °C, and VO2 and VCO2 were recorded over a 15 min period; data obtained over the last 10 min were used to calculate BMR. BMR estimation was based on Weir eq. [30]: BMR (kcal/min) = [3.9(VO2) + 1.1(VCO2)]. The value obtained was multiplied by 1440 in order to estimate BMR for 24 h (kcal/day). BMR was also calculated from the relationship with BM or FFM (kcal/kg/24 h). Additionally, mean values for the respiratory quotient (RQ), VO2 (L/min) and metabolic equivalent (MET) were also obtained, taking 1 MET to be equal to an expenditure of 3.5 mL O2/kg/min.

### Blood analysis

Blood extraction was carried out using a clinical routine laboratory protocol after 12 h of patients fasting. Patients were advised to restrain from perform exercise or consuming alcohol 24 h prior the test. Patients consumed food with lipid composition similar to that ingested during the months the study took place. Glucose concentrations, cholesterol and triglycerides levels were measured using a colorimetric enzyme assay method (CEPA® kits – MBiolog Diagnósticos Ltda.) as previously described [31].

### Genetic analysis

Total DNA was isolated from mononuclear cell present in periphery blood through a Ficol gradient. DNA was extracted from this fraction using cloroform-isoamyl alcohol method. DNA was quantified with a spectrophotometer reading absorbance at 260 nm. This DNA was used to analyze whether the patients carry the different polymorphism under study. The presence of FTO rs9939609 A/T was analyzed through restriction fragment length polymorphism (RFLP), where primers were F 5′- GGT TCC TTGCGA CTG CTG TGA AAT T ‘3 and R 5’ GCT TTT ATGCTC TCC CAC TC ‘3. After PCR, amplicons were subject to action of ApoI restriction enzyme and RFLP fragments were analyzed in agarose gel as previously reported [32]. Presence of PLIN4 11482G > A, polymorphism was analyzed using allelic discrimination assay with Taqman probes in an Abi Prism 7500© (Applied biosystems) as previously described [16]. Presence of ADBR3 trp64ARg polymorphism was also analyzed through RFLP technique. Primers used were Forward 5´-CGCCCAATACCGCCAACAC-3′ and reverse 5´-CCACCAGGAGTCCCATCACC-3′ and the resulting amplicon was digested with BstOI restriction enzyme as previously described [33].

### Statistic

Results are presented as mean ± SEM. We use SPSS© r22 to perform an analysis of covariance to find out if there were significant differences among measurements and the possible influence of different variables such as sex, polymorphism and disease. Pearson correlation test was used to discard possible influence of age (that is a continuous variable) in the results. A posteriori Tukey test run to analyze differences as considered appropriate. We considered a *p* < 0.05 as significant. We estimated simple size (n) by power analysis running the following parameters in G*power (V3.1.9.2). These parameters were type I and II errors of 5 and 95% respectively, and an effect size of 0.25. With these parameters the G*power estimated that the minimum sample size to find any significant differences should be a minimum of 210.

## Results

### Intervention effect in weight and basal metabolic rate, fat mass and visceral fat

**Fig. 1:**
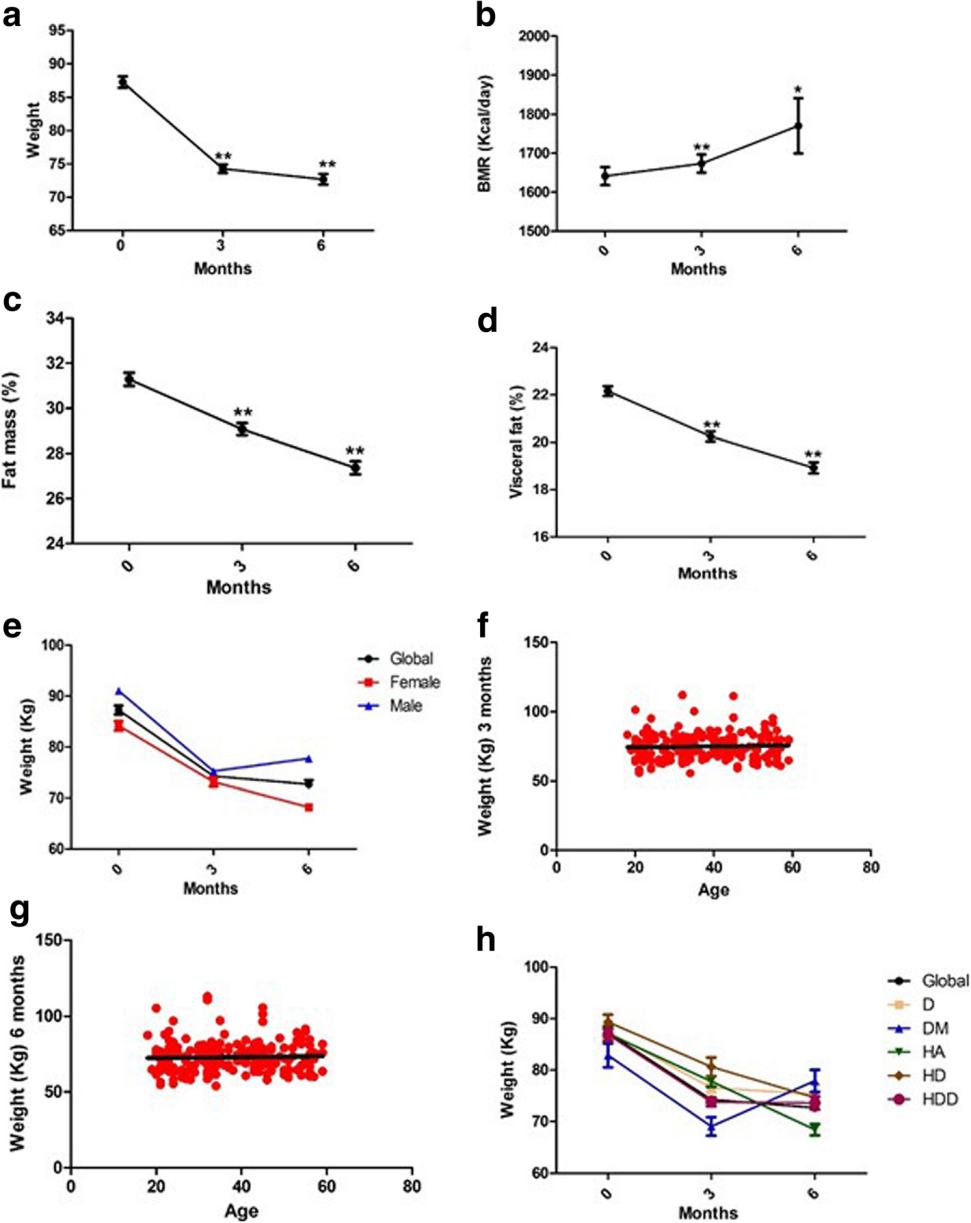
Effects of GC and GNN on body composition. Weight (**a**), Basal metabolic rate (**b**), Fat mass (**c**), Visceral Fat (**d**) and influence of sex (**e**), age − 3 months (**f**) and 6 months (**g**)- and presence of comorbidities (**h**) in weight reduction. D: Dyslipidemias, DM: Diabetes mellitus type 2, H: Hypertension, HD: Hypertension and Dyslipidemias, HDD: hypertension, dyslipidemias and DM2. *p < 0.05;**p < 0.01 vs treatment onset (0 months)

We first analyzed whether the treatment of people with overweight or obesity with GC and GNN had any effect of weight, metabolic basal rate, fat mass and visceral fat. We found a dramatic, highly significant and sustained reduction of weight at 3 and 6 months compared with the start of the study (Fig. 1a). This decrease was parallel to a highly significant and significant increase in metabolic basal rate in 3 months 6 months respectively (Fig. 1b). Weight loss was also related to a highly significant reduction of fat mass and visceral fat in 3 and 6 months (Fig. 1c). Weight decrease was independent of sex, age, or suffering from hypertension, DM2, dyslipidemias or their combinations (Fig. 1d-h). It is important to underline that there were no adverse effects reported by the patients or by medical staff in patient clinical examination.

### Treatment with GC and GNN produces changes in metabolism

**Fig. 2:**
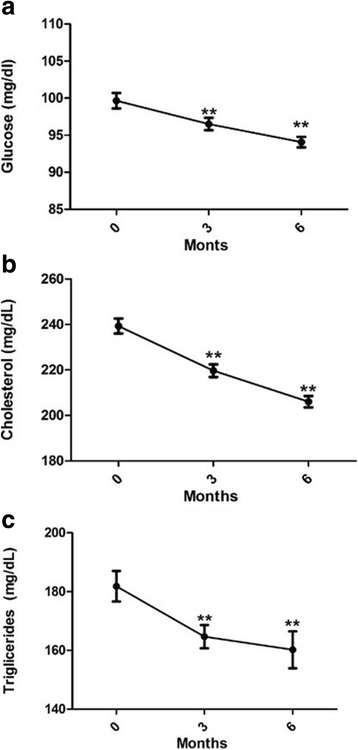
Effect of GC and GNN on level of metabolic markers. Glucose (**a**), Cholesterol (**b**) and Triglycerides (**c**). **p < 0.01 vs treatment onset (0 months)

**Fig. 3:**
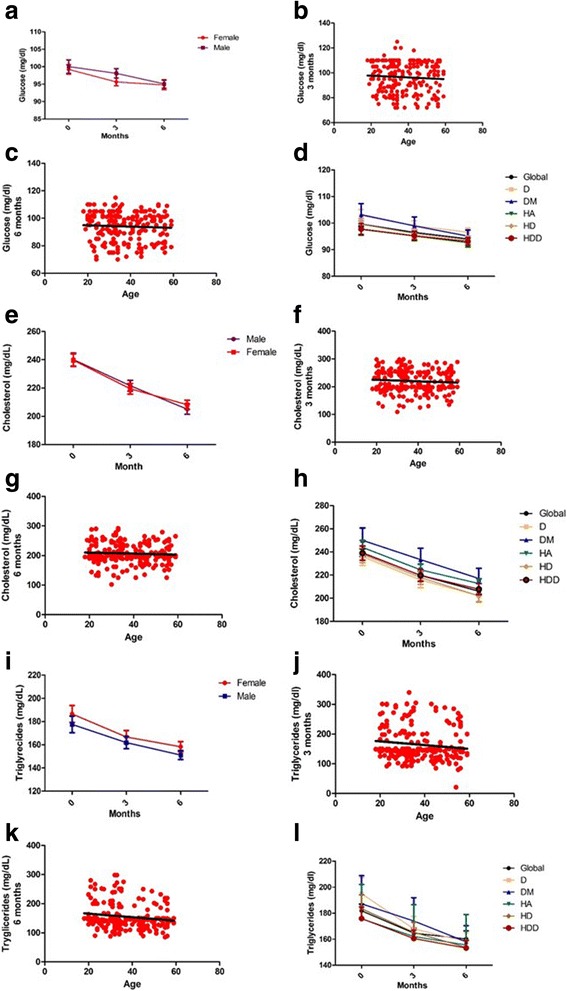
Effect of GC and GNN is independent of sex, age and comorbidities. After treatment levels of glucose is not influenced by sex (**a**), age − 3 months (**b**), 6 months (**c**), comorbidities (**d**). Treatment effects on cholesterol is not influenced by sex (**e**), age 3 months (**f**) and 6 months (**g**) or comorbidities (**h**). Also, GC and GNN effect on triglycerides level are not influenced by sex (**I**), age 3 months (**j**) and 6 months (**k**) or comorbidities (**l**). D: Dyslipidemias, DM: Diabetes mellitus type 2, H: Hypertension, HD: Hypertension and Dyslipidemias, HDD: hypertension, dyslipidemias and diabetes mellitus type 2

Regarding glucose level, we found that the treatments reduce it significantly at 3 and 6 months compared with basal levels (Fig. 2a). This reduction was also significant and sustained for cholesterol and triglycerides plasma levels (Fig. 2b, c). These changes in glucose, cholesterol and triglycerides levels, were not related to sex, age, or suffering from the different comorbidities (Fig. 3a-l).

### Presence of polymorphism affect weight reduction, mass fat and visceral fat

**Fig. 4:**
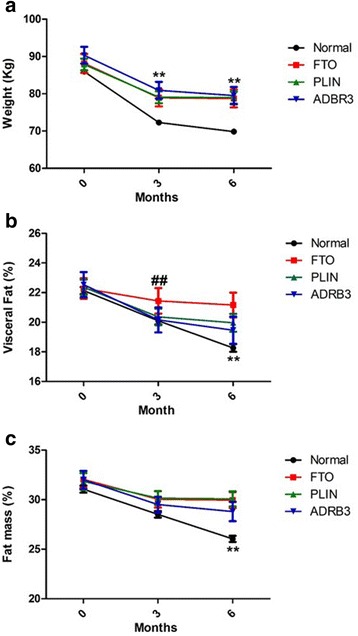
Effect of polymorphism in body composition after treatment with GC and GNN. Presence of polymorphism PLIN4 (11482G > **a**), FTO (rs9939609 A/T), and ADRB3 (Trp64Arg) attenuated response to GC and GNN treatment on weight (**a**), Visceral Fat (**b**) and Fat mass (**c**). **p < 0.05 vs all polymorphism presence. ^##^*p* < 0.05 FTO vs normal. PLIN: 11482G > A, FTO: rs9939609 A/T and ADRB3: Trp64Arg

**Fig. 5:**
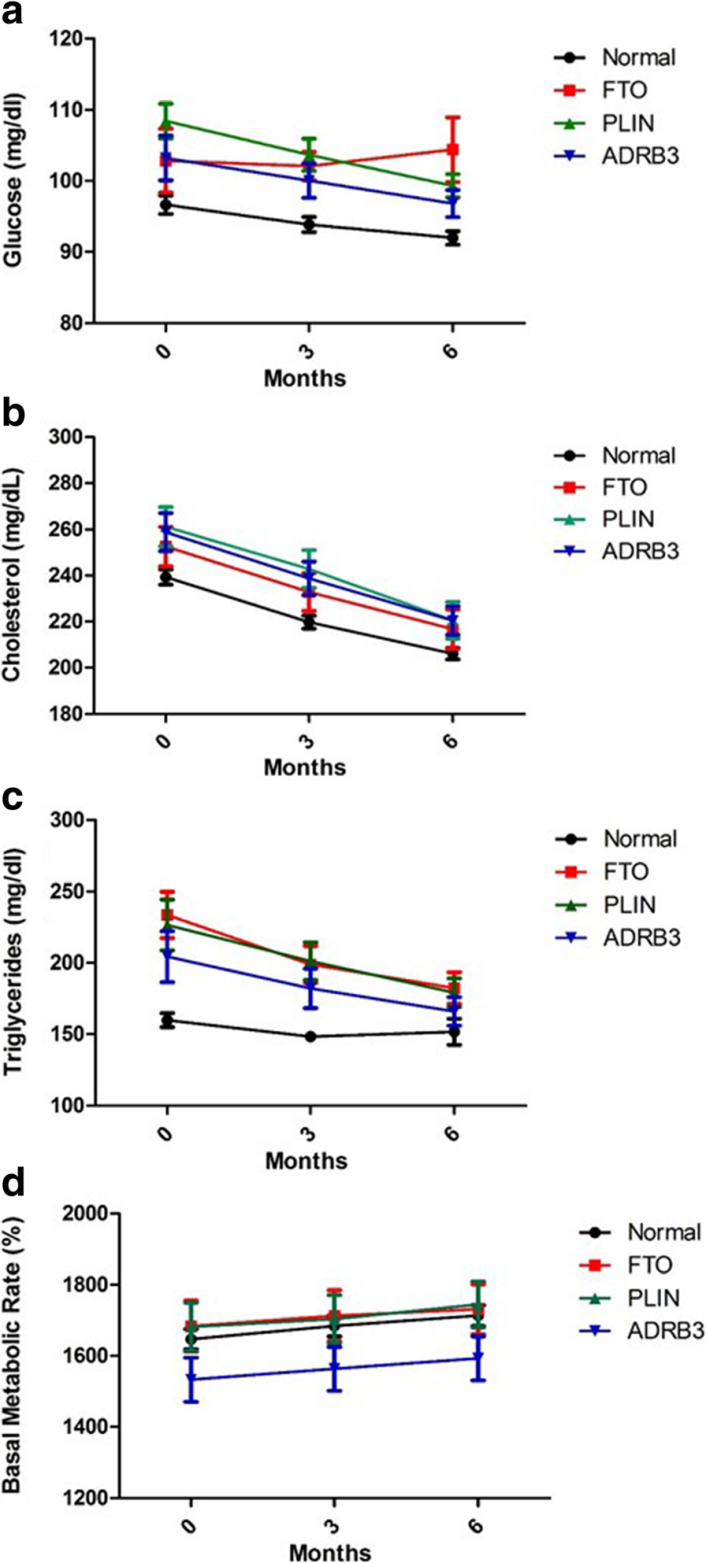
Influence of polymorphism after treatment with GC and GNN on metabolic markers levels. Glucose (**a**), Cholesterol (**b**), Triglycerides (**c**) and Basal metabolic rate (**d**). PLIN: 11482G > A, FTO: rs9939609 A/T and ADRB3: Trp64Arg polymorphism

We wanted to determinate If carry any of the three-studied polymorphism in the different analyzed parameter affected the result of treatment. Patients carriers any of the three polymorphism under study, shown an attenuated reduction in weight at 3 and 6 months (Fig. 4a). Besides, we have found that carriers of FTO polymorphism reduce significant less visceral fat than normal patients at 3 months whereas 6 months carriers of FTO (rs9939609 A/T), PLIN4 (11482G > A) and ABDR3 (Trp64Arg) polymorphisms reduce significantly less visceral fat than non-carriers (Fig. 4b). Regarding Fat mass (%), we found that patients carriers any of the three polymorphism reduce less fat mass than non-carriers subject at 6 months (Fig. 4c). On the other hand, either glucose, cholesterol, triglycerides, or BMR were not significantly affected by any of the studied polymorphism (Fig. 5a-d).

## Discussion

Here, we wanted to expand previous studies in which treatment with GC and GNN were combined to reduce obesity [31]. In this previous study, that was a double blinded randomized study with a placebo and treated group, it was reported that GC and GNN administration reduced cholesterol levels but without affecting anthropometric or calorimetric values. Now, we show that administration of a combined therapy of GC and GNN to 214 people with overweight or obesity and some associated comorbidities such us dyslipidemias, hypertension and DM2, reduce at 3 and 6 months the weight (14 and 16%), fat mass (7 and 12%), visceral fat (9 and 15%), glucose (3 and 6%), cholesterol (8 and 13%), triglycerides (10 and 15.3%) and increase BMR measured by indirect calorimetry (2 and 4%). Several reports support that GC administration reduces weight [34, 35] although other has not found enough evidence [36] or doubt about its long term effects because there are not reported on more than 12 months of administration [26]. These differences, even some reported minor side effects, might be related to the different compositions of available GC extracts and administered doses [37]. On the other hand, GNN has also proved to reduce weight [38] although other studies did no find such association [39]. These differences might be related to variability of patient inclusion criteria in each study, and as in the case of GC to the different composition of Glucomannan extracts used. Of note, it is important to underline that here, the combined treatment, is able to reduce weight, fat mass and visceral fat independently of sex, age and previous diseases without having aby adverse effects in the patients which makes this treatment suitable for a broad population segment.

On the other hand, it has been reported that success of obesity therapies has been influenced by individual variability mainly based on differences on life style and genetic background [20, 40]. For these reason, we also aim to study the influence of different polymorphism associated to obesity risk with a treatment with GC and GNN to reduce weight. Thus, the second objective or our work was to determine whether presence of different polymorphism might affect the result of GC and GNN combined treatment. When we analyzed the results by polymorphism effect, we found that there were an attenuated weight reduction, visceral fat and fat mass in carriers of FTO (rs9939609 A/T), PLIN4 (11482G > A) and ADRB3 (Trp64Arg) polymorphism at the two different times. Polymorphisms in *PLIN* and *ADRB3* use to produce alteration in lipolysis mechanism (i.e. decreasing it) [41] whereas treatment with GC and GNN have demonstrated to produce loss weight particularly increasing lipolysis [42]. Thus, it would be reasonable to hypothesize that a reduction in lipolysis rate in patients with these polymorphisms might have hampered the lipolysis effect trigger by GC and GNN influencing the outcome of the treatment.

*PLIN* gene codified perilipin 1 protein that is phosphorylated by protein kinasa A. Perilipin 1 phosphorylation facilitates the action of hormone sensitive lipase, an enzyme that play a key role in lipolysis. Several polymorphisms in this gene has been associated to obesity [41, 43]. Thus, patients carriers of *PLIN1* (6209 T > C; rs2289487) and *PLIN6* (14995A > T; rs1052700) polymorphism have shown to response better to anti-obesity treatment [16]. On the contrary, and in agreement with our results, those patients carriers of PLIN4 (11482G > A) response worse to weight loss treatments [44].

Regarding ADRB3, this receptor plays an important role in the lipolysis activated by catecholamine signaling. The change Trp64Arg produces that ADRB3 does not reach the maximal generation of cyclic AMP upon activation and thus reduce lipolysis [45]. Accordingly, in our study, carriers of this polymorphism did not reduce weight, fat mas and visceral fat at the same rate that non-carriers subjects. However, although levels of triglycerides and cholesterol were increase compared with non-carrier patients, these levels did not reach a statistical significance.

On the other hand, FTO polymorphism rs9939609 is associated to a low satiety feeling after meals rather than a regulation of basal metabolic rate [45]. Here, we found that patients with this polymorphism does not reduce weight, visceral fat and fat mass as normal patients. These results might be explained because these patients has a tendency to increase calorie intake, hindering treatments effects. Considering that presence of the different polymorphism studied in this work influence the grade of losing weight, fat mass and visceral mass, it would be of the utmost importance to previously known the genetic background of each subject to adjust the treatment (perhaps increasing dose of GC or GNN) to obtain a better outcome.

## Conclusions

Taken together these data, we conclude that treatment with GC and GNN of people with overweight or obesity with different sex, age and affected by difference metabolic diseases decrease weight, fat mass, visceral fat, glucose, triglycerides and cholesterol levels together with an increasing basal metabolic rate without having any adverse effect. Given that presence of different polymorphism such as PLIN4 (11482G > A), FTO (rs9939609 A/T) and ADRB3 Trp64Arg might hamper this beneficial effects, a patient genetic study focus on the presence of these polymorphisms might be advisable previously to establish a treatment and thus get a better prediction of response treatment.

## Additional files

Additional file 1: TREND Statement Checklist. (PDF 1254 kb)

Additional file 2: TRENDS flowchart. (JPEG 965 kb)

## Abbreviations

ADRB3: β-adrenergic receptor 3
BMI: Body Mass index
FTO: Fat mass and obesity-associated
GC: Garcina *cambogia*
GNN: Glucomannan

## References

[1] WHO (2012). Obesity and overweight.

[2] Finucane MM, Stevens GA, Cowan MJ, Danaei G, Lin JK, Paciorek CJ (2011). National, regional, and global trends in body-mass index since 1980: systematic analysis of health examination surveys and epidemiological studies with 960 country-years and 9·1 million participants. Lancet (London, England).

[3] Must A, McKeown NM. The Disease Burden Associated with Overweight and Obesity. Endotext. 2000.

[4] Calle EE, Rodriguez C, Walker-Thurmond K, Thun MJ (2003). Overweight, obesity, and mortality from cancer in a prospectively studied cohort of U.S. adults. N Engl J Med.

[5] Daniels SR, Arnett DK, Eckel RH, Gidding SS, Hayman LL, Kumanyika S (2005). Overweight in children and adolescents: pathophysiology, consequences, prevention, and treatment. Circulation.

[6] Garrow JS, Webster J (1985). Quetelet’s index (W/H2) as a measure of fatness. Int J Obes.

[7] Bell CG, Walley AJ, Froguel P (2005). The genetics of human obesity. Nat Rev Genet.

[8] McAllister EJ, Dhurandhar NV, Keith SW, Aronne LJ, Barger J, Baskin M (2009). Ten putative contributors to the obesity epidemic. Crit Rev Food Sci Nutr.

[9] Madrigano J, Baccarelli A, Wright RO, Suh H, Sparrow D, Vokonas PS (2010). Air pollution, obesity, genes and cellular adhesion molecules. Occup Environ Med.

[10] Cummings DE, Schwartz MW (2003). Genetics and pathophysiology of human obesity. Annu Rev Med.

[11] Wardle J, Carnell S, Haworth CM, Plomin R (2008). Evidence for a strong genetic influence on childhood adiposity despite the force of the obesogenic environment. Am J Clin Nutr.

[12] Kaur Y, de Souza RJ, Gibson WT, Meyre D (2017). A systematic review of genetic syndromes with obesity. Obes Rev.

[13] Gerken T, Girard CA, Tung Y-CL, Webby CJ, Saudek V, Hewitson KS (2007). The obesity-associated FTO gene encodes a 2-oxoglutarate-dependent nucleic acid demethylase. Science.

[14] Qi Q, Kilpelainen TO, Downer MK, Tanaka T, Smith CE, Sluijs I (2014). FTO genetic variants, dietary intake and body mass index: insights from 177 330 individuals. Hum Mol Genet.

[15] Frayling TM, Timpson NJ, Weedon MN, Zeggini E, Freathy RM, Lindgren CM (2007). A common variant in the FTO gene is associated with body mass index and predisposes to childhood and adult obesity. Science NIH Public Access.

[16] Qi L, Corella D, Sorlí JV, Portolés O, Shen H, Coltell O (2004). Genetic variation at the perilipin (PLIN) locus is associated with obesity-related phenotypes in white women. Clin Genet.

[17] Mottagui-Tabar S, Rydén M, Löfgren P, Faulds G, Hoffstedt J, Brookes AJ (2003). Evidence for an important role of perilipin in the regulation of human adipocyte lipolysis. Diabetologia.

[18] Brasaemle DL, Rubin B, Harten IA, Gruia-Gray J, Kimmel AR, Londos C (2000). Perilipin a increases triacylglycerol storage by decreasing the rate of triacylglycerol hydrolysis. J Biol Chem.

[19] Clément K, Vaisse C, Manning BS, Basdevant A, Guy-Grand B, Ruiz J (1995). Genetic variation in the beta 3-adrenergic receptor and an increased capacity to gain weight in patients with morbid obesity. N Engl J Med.

[20] MacLean PS, Wing RR, Davidson T, Epstein L, Goodpaster B, Hall KD (2015). NIH working group report: innovative research to improve maintenance of weight loss. Obesity.

[21] Preuss HG, Bagchi D, Bagchi M, Rao CVS, Dey DK, Satyanarayana S (2004). Effects of a natural extract of (−)-hydroxycitric acid (HCA-SX) and a combination of HCA-SX plus niacin-bound chromium and Gymnema Sylvestre extract on weight loss. Diabetes, Obes. Metab.

[22] Ramos R, Sanz S, Aguilar S (1995). Extract of Garcinia cambogia in the control of obesity. Invest Med Int.

[23] Toromanyan E, Aslanyan G, Amroyan E, Gabrielyan E, Panossian A (2007). Efficacy of Slim339 in reducing body weight of overweight and obese human subjects. Phytother Res.

[24] Girola M, De Bernardi M, Contos S. Dose effect in lipidlowering activity of a new dietary integrator (Chitosan, Garcinia cambogia extract, and chrome). Acta Toxicol Ther. 1996;17:25–40.

[25] Birketvedt G, Shimshi M, Thom E, Florholmen J (2005). Experiences with three different fiber supplements in weight reduction. Med Sci Monit.

[26] Márquez F, Babio N, Bulló M, Salas-Salvadó J (2012). Evaluation of the safety and efficacy of hydroxycitric acid or Garcinia cambogia extracts in humans. Crit Rev Food Sci Nutr.

[27] Lyon MR, Reichert RG (2010). The effect of a novel viscous polysaccharide along with lifestyle changes on short-term weight loss and associated risk factors in overweight and obese adults: an observational retrospective clinical program analysis. Altern Med Rev.

[28] Cairella M, Marchini G (1995). [evaluation of the action of glucomannan on metabolic parameters and on the sensation of satiation in overweight and obese patients]. Clin. Ter.

[29] Deurenberg P, van der Kooy K, Leenen R, Weststrate JA, Seidell JC (1991). Sex and age specific prediction formulas for estimating body composition from bioelectrical impedance: a cross-validation study. Int J Obes.

[30] WEIR JBDB. New methods for calculating metabolic rate with special reference to protein metabolism. J Physiol 1949;109:1–9. 10.1113/jphysiol.1949.sp004363 PMC1392602 15394301

[31] Vasques CAR, Rossetto S, Halmenschlager G, Linden R, Heckler E, Fernandez MSP (2008). Evaluation of the pharmacotherapeutic efficacy of *Garcinia cambogia* plus *Amorphophallus konjac* for the treatment of obesity. Phyther Res.

[32] Eynon N, Nasibulina ES, Banting LK, Cieszczyk P, Maciejewska-Karlowska A, Sawczuk M (2013). The FTO A/T polymorphism and elite athletic performance: a study involving three groups of European athletes. PLoS One.

[33] Widén E, Lehto M, Kanninen T, Walston J, Shuldiner AR, Groop LC (1995). Association of a Polymorphism in the β _3_ -adrenergic–receptor gene with features of the insulin resistance syndrome in Finns. N Engl J Med.

[34] Sun N-N, Wu T-Y, Chau C-F (2016). Natural dietary and herbal products in anti-obesity treatment. Molecules.

[35] Semwal RB, Semwal DK, Vermaak I, Viljoen A (2015). A comprehensive scientific overview of Garcinia cambogia. Fitoterapia.

[36] Heymsfield SB, Allison DB, Vasselli JR, Pietrobelli A, Greenfield D, Nunez C (1998). Garcinia cambogia (hydroxycitric acid) as a potential antiobesity agent: a randomized controlled trial. JAMA.

[37] Chuah LO, Ho WY, Beh BK, Yeap SK (2013). Updates on Antiobesity effect of Garcinia origin (−)-HCA. Evid Based Complement Alternat Med.

[38] Sood N, Baker WL, Coleman CI (2008). Effect of glucomannan on plasma lipid and glucose concentrations, body weight, and blood pressure: systematic review and meta-analysis. Am J Clin Nutr.

[39] Onakpoya I, Posadzki P, Ernst E (2014). The efficacy of glucomannan supplementation in overweight and obesity: a systematic review and meta-analysis of randomized clinical trials. J Am Coll Nutr.

[40] Bray GA, Wadden TA (2015). Improving long-term weight loss maintenance: can we do it?. Obesity (Silver Spring).

[41] Luglio HF, Sulistyoningrum DC, Susilowati R (2015). The role of genes involved in lipolysis on weight loss program in overweight and obese individuals. J Clin Biochem Nutr.

[42] Sethi A (2011). A review on “Garciniacambogia – a weight controll in gagent”. IJPRD.

[43] Smith CE (2012). Ordov?S JM. Update on perilipin polymorphisms and obesity. Nutr. Rev.

[44] Corella D, Qi L, Sorlí JV, Godoy D, Portolés O, Coltell O (2005). Obese subjects carrying the 11482G>a polymorphism at the perilipin locus are resistant to weight loss after dietary energy restriction. J Clin Endocrinol Metab.

[45] Candelore MR, Deng L, Tota LM, Kelly LJ, Cascieri MA, Strader CD (1996). Pharmacological characterization of a recently described human beta 3-adrenergic receptor mutant. Endocrinology.

