# Supplementary figures and images for: Long-term effects of Garcinia cambogia/Glucomannan on weight loss in people with obesity, PLIN4, FTO and Trp64Arg polymorphisms
Source: BMC Complement Altern Med. 2018 Jan 24;18:26. doi: 10.1186/s12906-018-2099-7 (PMC5781311; doi:10.1186/s12906-018-2099-7)

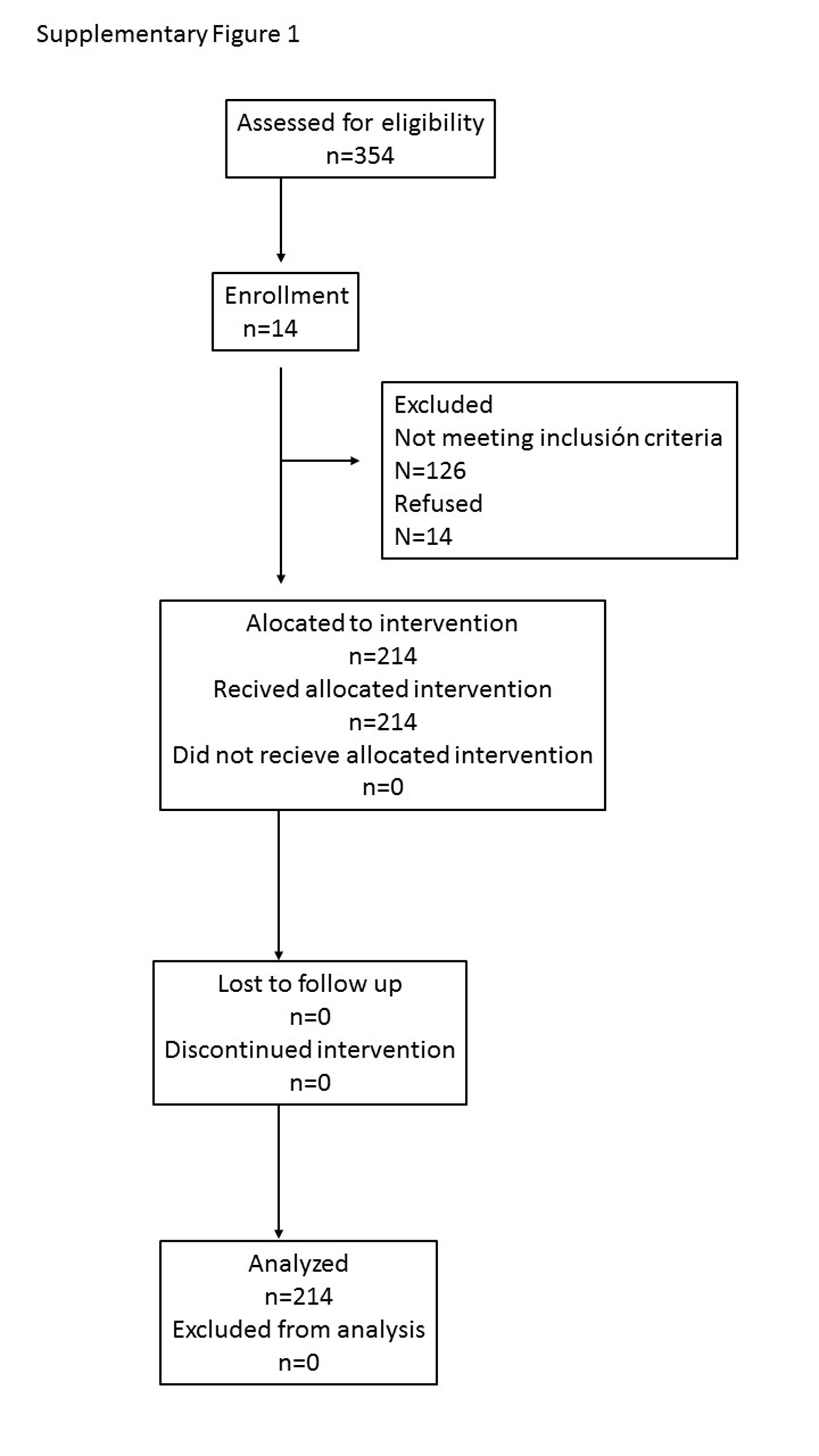

Supplement: Supplementary file 2 — TRENDS flowchart. (JPEG 965 kb) [file 12906_2018_2099_MOESM2_ESM.jpg]
